# Pathogenicity Differentiation of *Fusarium* spp. Causing Fusarium Basal Rot and Wilt Disease in *Allium* spp.

**DOI:** 10.3390/pathogens13070591

**Published:** 2024-07-16

**Authors:** Kosei Sakane, Takashi Ueno, Masayoshi Shigyo, Kazunori Sasaki, Shin-ichi Ito

**Affiliations:** 1 The United Graduate School of Agricultural Sciences, Tottori University, Tottori 680-8553, Japan; necchange999@outlook.jp; 2 Graduate School of Sciences and Technology for Innovation, Yamaguchi University, Yamaguchi 753-8515, Japan; b009gd@yamaguchi-u.ac.jp (T.U.); shigyo@yamaguchi-u.ac.jp (M.S.); 3 Research Center for Thermotolerant Microbial Resources (RCTMR), Yamaguchi University, Yamaguchi 753-8515, Japan

**Keywords:** *Fusarium* species, *Allium fistulosum* L., *Allium cepa* L., pathogenicity, *SIX* gene, host specificity

## Abstract

Here, 12 *Fusarium* strains, previously described as *F*. *oxysporum* f. sp. *cepae* (*Foc*), were examined via multi-locus sequencing of calmodulin (*cmdA*), RNA polymerase II second largest subunit (*rpb2*), and translation elongation factor 1-alpha (*tef1*), to verify the taxonomic position of *Foc* in the newly established epitype of *F*. *oxysporum*. The strains in this study were divided into two clades: *F*. *nirenbergiae* and *Fusarium* sp. To further determine the host specifications of the strains, inoculation tests were performed on onion bulbs and Welsh onion seedlings as potential hosts. Four strains (AC145, AP117, Ru-13, and TA) isolated from diseased onions commonly possessed the *secreted in xylem* (*SIX*)-*3*, *5*, *7*, *9*, *10*, *12*, and *14* genes and were pathogenic and highly aggressive to onion bulbs, whereas all strains except for one strain (AF97) caused significant inhibition of Welsh onion growth. The inoculation test also revealed that the strains harboring the *SIX9* gene were highly aggressive to both onion and Welsh onion and the gene was expressed during infection of both onions and Welsh onions, suggesting the important role of the *SIX9* gene in pathogenicity. This study provides insights into the evolutionary pathogenicity differentiation of *Fusarium* strains causing Fusarium basal rot and wilt diseases in *Allium* species.

## 1. Introduction

The *Fusarium oxysporum* species complex (FOSC) contains cosmopolitan, soil-borne, filamentous fungi infecting over 120 economically important crops [1]. *F*. *oxysporum* species are divided into “*formae speciales*” according to host specificity and further subdivided into “race” based on cultivar-specific pathogenicity, which renders *F*. *oxysporum* taxonomy complicated [2]. To classify the *F*. *oxysporum* species, the genetic similarity of the gene loci, including the internal transcribed spacer (ITS) region, mitochondrial small subunit, RNA polymerase 1 and 2 (*rpb1* and *rpb2*), and translation elongation factor 1-alpha (*tef1*), has been investigated [3,4,5,6]. However, identification of *Fusarium* species using a single locus is insufficient to distinguish between different *Fusarium* species [7]. The epitype of *F*. *oxysporum* has been established based on multiple loci, including *cmdA*, *rpb2*, and *tef1* genes, using phylogenetic analysis to correct the taxonomic classification of *F*. *oxysporum* [8]. Moreover, phylogenetic analysis using multi-locus sequences has been used to prevent the incorrect identification of *F*. *oxysporum* [3,9,10].

Among *F*. *oxysporum* strains, *F*. *oxysporum* f. sp. *cepae* (*Foc*) is the causative agent of Fusarium basal rot and wilt disease in onions (*Allium cepa* L.) and Welsh onions (*A*. *fistulosum* L.), also known as Japanese bunching onion, which negatively affect onion and Welsh onion production worldwide [11,12,13,14]. Therefore, many studies have focused on the pathogenicity-related factors in *Foc* to understand the mechanisms by which *Foc* infects onion and Welsh onion. *Secreted in xylem* (*SIX*) genes promote colonization and host infection in *F*. *oxysporum* [15,16,17,18]. *SIX* genes are frequently found on a unique chromosome, known as the pathogenicity chromosome, in *F*. *oxysporum*. Pathogenicity chromosomes are necessary for pathogenicity to determine the host range and are transferable among strains [17,19,20,21]. *F*. *oxysporum* has been reported to have evolutionally acquired the virulence factor via chromosome transfer to exert host-specific pathogenicity toward individual plant species. Likewise, it has also been reported that the pathogenicity differentiation of *F*. *oxysporum* is correlated with individual phylogenetic evolution [4,5].

To date, *SIX2*, *3*, *5*, *7*, *9*, *10*, *12*, and *14* gene homologs have been identified in the genome of onion- and Welsh onion-infecting *Foc* strains [22,23]. In the onion-infecting *Foc*, the *SIX3*, *5*, *7*, *9*, *10*, *12*, and *14* genes are located on a single pathogenicity chromosome that is required for full pathogenicity toward onion [22]. Additionally, the *SIX3*, *5*, and *9* genes are highly upregulated during *Foc* infection of onion [23]. *Foc* strains showing high expression levels of the *SIX9* gene are relatively more aggressive toward onions than those showing low expression levels of the *SIX9* gene [24]. Furthermore, the *SIX2* and *SIX9* genes are located on a 3 Mb chromosome and expressed in Welsh onion-infecting *Foc* [25]. Pathogenicity-related factors have been extensively elucidated in *Foc* strains; however, the reasons for their host specificity toward onions and Welsh onions remain unknown.

In this study, we aimed to clarify the taxonomic position of *Fusarium* strains causing basal rot and wilt disease in onions and Welsh onions and examine their host specificity to onions and Welsh onions.

## 2. Materials and Methods

### 2.1. Fungal Strains and Plant Materials

The fungal strains previously identified as *F*. *oxysporum* based on morphology and ribosomal DNA ITS region sequences used in this study are listed in Table 1. The onion cultivar Kitamomiji 2000 (Shippou Co., Ltd., Kagawa, Japan) and Welsh onion cultivar Fuyuhiko (Nakahara Seed Product Co., Ltd., Fukuoka, Japan) were used in this study.

### 2.2. DNA Isolation, Polymerase Chain Reaction (PCR), and Sequencing

Total genomic DNA was extracted from each fungal strain cultured in a potato dextrose broth (PDB) medium (Becton, Dickinson and Company, Franklin Lakes, NJ, USA) for five days at 25 °C, and the fungal mycelia were harvested by filtering the broth through a sterile filter paper (Advantec, Tokyo, Japan). Fungal DNA was extracted from the harvested mycelia using the Dr. GenTLE (from yeast) High Recovery Kit (TaKaRa, Osaka, Japan). For PCR and sequence analysis of the *SIX* genes, we used a 20 µL reaction mixture containing 10 µL of Quick Taq HS Dye Mix (Toyobo, Osaka, Japan), 0.2 µM of designated primer, and 20 ng of genomic DNA. Thermal cycling conditions consisted of 2 min at 94 °C, followed by 30 cycles of 30 s at 94 °C, 30 s at 55–68 °C, and 1 min at 68 °C. PCR products were separated via electrophoresis on a 1.2% (*w*/*v*) agarose gel (NIPPON GENE, Tokyo, Japan), stained with ethidium bromide, and visualized under UV light. For sequencing analysis, the PCR amplicon was purified via ethanol precipitation and labeled with the BigDye Terminator v3.1 Cycle sequencing Kit (Applied Biosystems, Foster City, CA, USA). The labeled DNA amplicons were sequenced using the ABI 3500xL genetic analyzer (Applied Biosystems). All primers used in this study are listed in Table S1. The sequences of calmodulin (*cmdA*), RNA polymerase II second largest subunit (*rpb2*), and translation elongation factor 1 (*tef1*) of each strain used in this study were deposited into the DNA Data Bank of Japan (https://www.ddbj.nig.ac.jp/index-e.html: accessed on 18 January 2024).

### 2.3. Phylogenetic Analysis

A multi-locus phylogenetic tree was constructed using the maximum likelihood method. The sequences of individual loci, including *cmdA*, *rpb2*, and *tef1*, were aligned using Clustal 2.1 [26]. The phylogenetic tree was constructed using MEGA 11 [27].

### 2.4. Onion Bulb Inoculation Test

Each strain was grown on potato dextrose agar (PDA) medium and incubated in a growth chamber at 25 °C for five days. Onion bulbs were surface-sterilized with 0.05% NaOCl for 3 min. Then, the central basal part of the sterilized onion bulbs was hollowed out with a 5 mm cork borer. The edge of the colony was also hollowed out with a 5 mm cork borer and embedded in the hollowed basal part of the sterilized onion bulbs. A planar PDA medium plug was embedded in the basal tissue of hollowed-out onions as a control. The inoculated onion bulbs were placed inside a plastic bag with a wet paper towel and incubated at 25 °C in a temperature-controlled room. After three weeks, symptoms in the inoculated onion bulbs were observed. Symptomatic areas, including mycelia and brown discoloration regions, were manually captured and estimated from the photographs using the Image J software (ver. 1.53) [28]. All tests were conducted with at least three samples per iteration. All experiments were repeated twice.

### 2.5. Welsh Onion Seedling Inoculation Test

Fungal strains were cultured in the PDB medium for seven days in a growth chamber at 25 °C, with shaking at 120 rpm, and subsequently filtered through three layers of sterilized gauze to collect the spores for inoculation. The spores were rinsed once with sterile water. The number of spores in the suspension was determined using a hemocytometer (Erma Inc., Saitama, Japan) and adjusted to a concentration of 1 × 10^4^ spores/mL. Welsh onion seeds were surface-disinfected with 0.05% NaOCl for 10 min and rinsed with water for 10 min. The disinfected seeds were inoculated with a spore suspension (1 × 10^4^ spores/mL) of each fungal strain or sterilized water (as a control) for 1 h. After treatment, the seven inoculated Welsh onion seeds were sown into plastic pots containing a 4:1 sand/compost mixture and incubated in a growth chamber at 25 °C under a 16 h light/8 h dark photoperiod. Finally, the lengths of the Welsh onion seedlings were measured 21 d post-inoculation, and the average length of the leaves was calculated.

### 2.6. RNA Extraction and Reverse Transcriptaion (RT)-PCR

For RT-PCR, RNA was extracted from the Welsh onion roots and onion bulbs inoculated with each strain at 7 or 21 d post-inoculation using Sepasol-RNA I Super G (Nacalai Tesque, Kyoto, Japan). Then, the total RNA containing genomic DNA was treated with DNase I (TaKaRa) to remove the genomic DNA. PrimeScript RT-PCR Kit (TaKaRa) was used to synthesize cDNA, following the manufacturer’s instructions. For the RT-PCR of the *SIX* genes, we used a 20 µL reaction mixture containing 10 µL of Quick Taq HS Dye Mix (Toyobo), 0.2 µM of designated primer, and 1 µL of cDNA. Thermal cycling conditions consisted of 2 min at 94 °C, followed by 30 cycles of 30 s at 94 °C, 30 s at 55 °C, and 1 min at 68 °C. PCR products were separated via electrophoresis on a 1.2% (*w*/*v*) agarose gel (NIPPON GENE), stained with ethidium bromide, and visualized under UV light.

### 2.7. Statistical Analyses

Experimental data are represented as the standard error of the mean. Statistically significant differences were determined using one-way analysis of variance followed by Tukey’s honest significant difference post hoc test.

## 3. Results

### 3.1. Verification of the Taxonomic Position of Foc Strains

**Figure 1:**
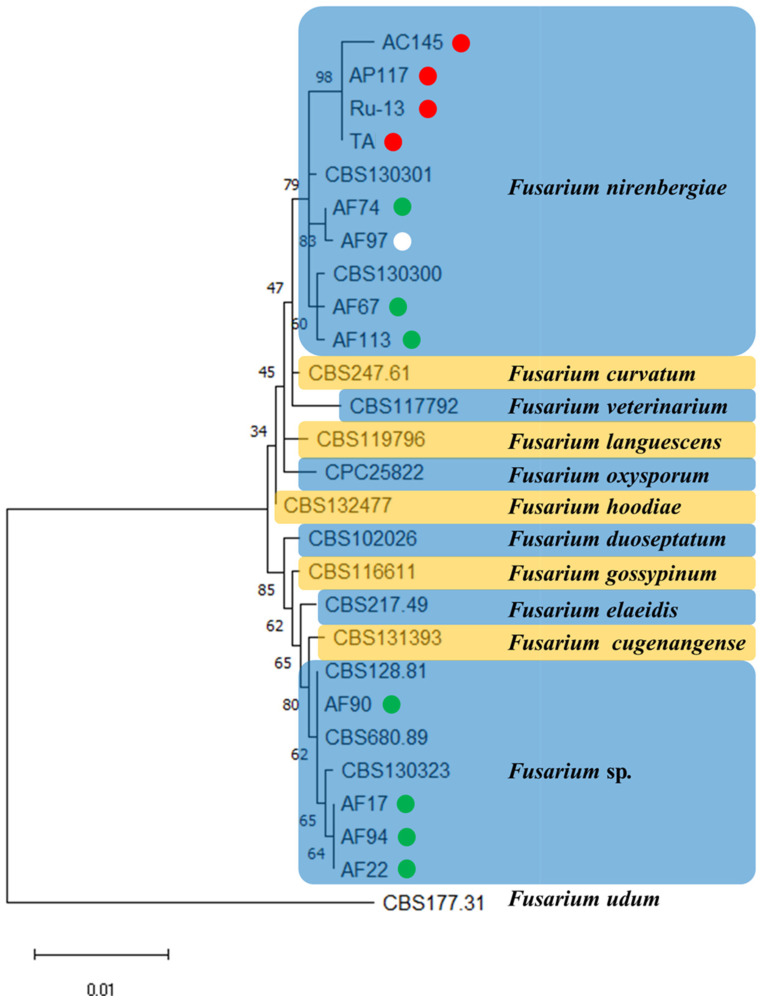
A maximum likelihood (ML) phylogram inferred from the sequences of calmodulin (*cmdA*), RNA polymerase II second largest subunit (*rpb2*), and translation elongation factor 1 (*tef1*). The numbers on the branches indicate the percentages of congruent clusters in 1000 bootstrap trials. The different color circles next to each strain name in the phylogenetic tree show the pathogenicity and host specificity. Red, green, and white circles indicate pathogenic strain toward onion and Welsh onion (Red circle), pathogenic strain toward Welsh onion (Green circle), and non-pathogenic strain toward onion and Welsh onion (White circle), respectively.

To verify the taxonomic position of strains belonging to the genus Fusarium (previously described as *Foc*) in the newly established epitype of *F. oxysporum*, we conducted phylogenetic analysis of the combined sequences of the *cmdA*, *rpb2*, and *tef1* genes of each strain. Based on the results of phylogenetic analysis, eight strains (AC145, AP117, Ru-13, TA, AF67, AF74, AF97, and AF113) were grouped into a clade of *F. nirenbergiae*, whereas four strains (AF17, AF22, AF90, and AF94) were grouped into a clade of *Fusarium* sp. (Figure 1).

### 3.2. Variation of SIX Gene Possession and Gene Expression Profiles

Pathogenicity is associated with the presence of *SIX* genes in *F*. *oxysporum*. Here, we verified the presence of *SIX* genes using PCR to assess the diversity of *SIX* genes in the strains isolated from onions and Welsh onions. PCR analysis revealed that the strains (AC145, AP117, Ru-13, and TA) isolated from onions commonly harbored the *SIX3*, *5*, *7*, *9*, *10*, *12*, and *14* genes. In contrast, the strains isolated from Welsh onions (AF17, AF22, AF90, and AF94) commonly harbored the *SIX2* and *SIX9* genes. Notably, AF74 possessed only the *SIX2* gene, and AF113 possessed only the *SIX9* gene. Moreover, there were no *SIX* genes in the genome of AF67 or AF97 (Table 1).

*SIX3*, *5*, and *9* levels are highly upregulated in onion-infecting *Fusarium* strains, whereas the *SIX2* and *SIX9* genes are expressed in Welsh onion-infecting *Fusarium* strains, suggesting these *SIX* genes are involved with the pathogenicity of the strains toward their respective hosts [24,26]. Here, we investigated *SIX3*, *5*, and *9* gene expression in the *Fusarium* strains isolated from onions and *SIX2* and *9* gene expression in the *Fusarium* strains isolated from Welsh onions during infection. Gene expression analysis revealed that the *Fusarium* strains isolated from onions expressed *SIX3*, *5*, and *9* not only during onion infection but also during Welsh onion infection. In contrast, *Fusarium* strains isolated from Welsh onions only expressed *SIX9* during both onion and Welsh onion infections. Notably, gene expression of *SIX2* was dependent on the microbial strain and host (Figure S1).

### 3.3. Inoculation Tests in Onion Bulbs and Welsh Onion Seedlings

**Figure 2:**
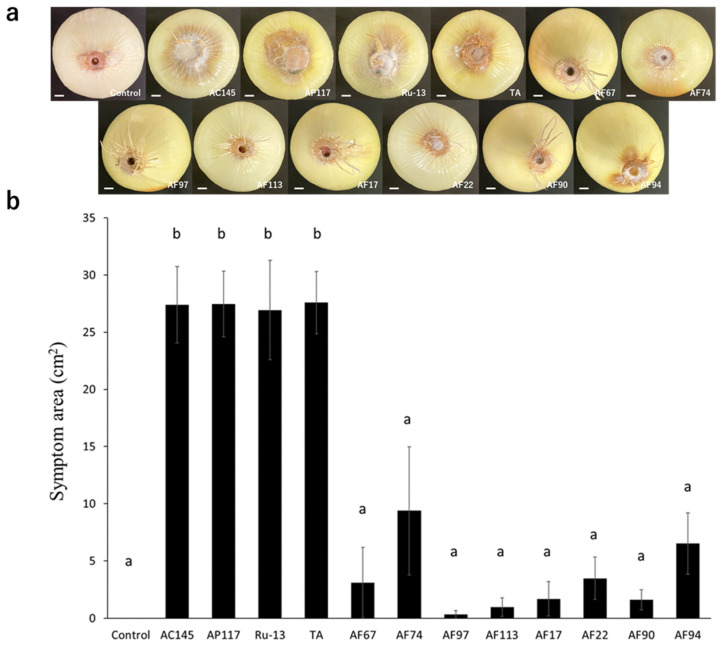
Onion bulb inoculation test. (**a**) Representative photograph of the onion bulb inoculation test with the *Fusarium* species strains used in this study. The white bar indicates the 1 cm scale bar. (**b**) Evaluation of symptom area in inoculated onion bulbs. The results of two independent experiments are combined. Statistically significant differences at *p* < 0.05 were analyzed via one-way analysis of variance (ANOVA) followed by Tukey’s honest significant difference (HSD) post hoc test and different letters indicate significant differences. All data are presented as the standard error of the mean (*n* = 6).

**Figure 3:**
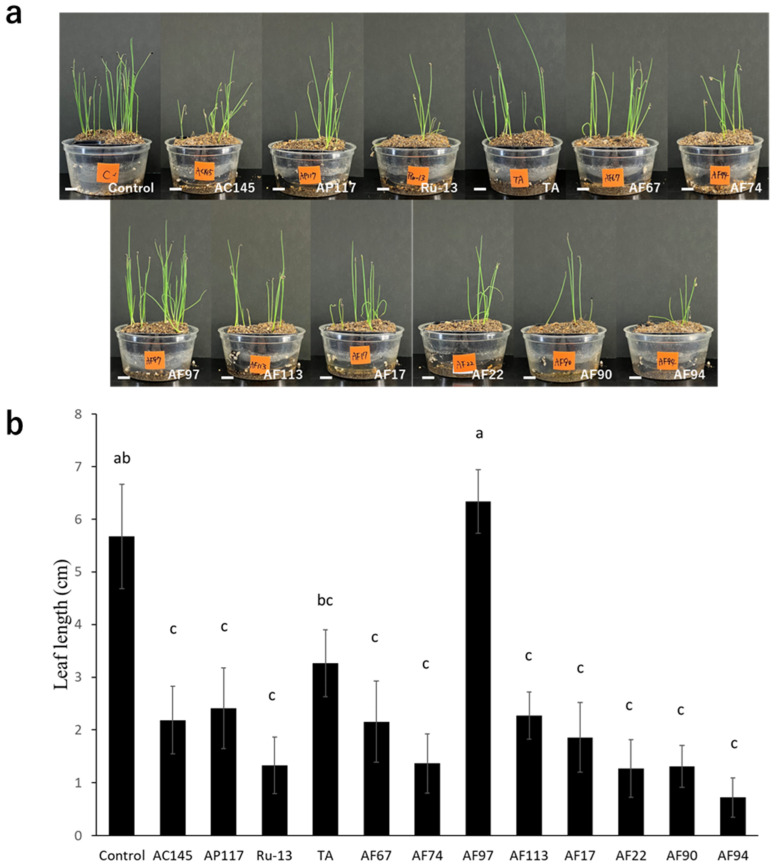
Welsh onion seedling inoculation test. (**a**) Representative photograph of Welsh onion seedling inoculation test with the *Fusarium* species strains used in this study. The white bar indicates the 1 cm scale bar. (**b**) Evaluation of average leaf length of the Welsh onion seedling. The results of two independent experiments are combined. Statistically significant differences at *p* < 0.05 were analyzed via one-way ANOVA followed by Tukey’s HSD post hoc test and different letters indicate significant differences. All data are presented as the standard error of the mean (*n* = 49).

To determine the pathogenicity toward onions, inoculation tests were performed on onion bulbs. Notably, all strains (AC145, AP117, Ru-13, and TA) isolated from onions caused severe disease symptoms in the onion bulbs, whereas the strains (AF17, AF22, AF67, AF74, AF90, AF94, AF97, and AF113) isolated from Welsh onions caused significantly fewer disease symptoms in the onion bulbs (Figure 2). Moreover, the symptoms in onion bulbs caused by the strains AF17, AF22, AF67, AF74, AF90, AF94, AF97, and AF113 were comparable to those observed in the control, with no significant difference. The inoculation test of Welsh onion seedlings revealed that all strains, except AF97, caused significant inhibition of Welsh onion growth (Figure 3).

## 4. Discussion

Plants are cultivated globally to meet the human food demand. However, cultivated plants are at risk of infection by pathogenic fungi, bacteria, and viruses [29,30,31]. Furthermore, due to global climate change caused by global warming, plants and plant pathogenic organisms are shifting to more ideal places than harsh environments to continue their life cycle [32]. Therefore, precise identification of plant pathogenic organisms and understanding of their host specificity are important for sustainable crop production and disease management.

In this study, the taxonomic position of the 12 *Fusarium* strains causing Fusarium basal rot and wilt disease in onions and Welsh onions was clarified. Based on the phylogenetic analysis of the combined sequences of *cmdA*, *rpb2*, and *tef1* loci, the strains were divided into *F*. *nirenbergiae* and *Fusarium* sp. clades. Notably, no one strain was identified as *F*. *oxysporum* sensu stricto, although all analyzed strains were previously identified as *F*. *oxysporum* f. sp. *cepae*. Presumably, the special form name can be linked to the accessory chromosome that contains genes of pathogenicity-related factors determining the host range and can be transferred via horizontal gene or chromosome transfer [8]. This leads to speculations about the transfer of pathogenicity-related factors among the *Fusarium* strains isolated from onions and Welsh onions.

To promote host infection, plant pathogenic organisms secrete effector proteins into the host tissue. In the *Fusarium* fungi, SIX proteins, known as effectors, secreted into xylem tissue have been broadly found [33,34]. Therefore, *SIX* gene possession analysis was conducted on the strains isolated from onions and Welsh onions. As a result of the *SIX* gene possession analysis using PCR, the strains (AC145, AP117, Ru-13, and TA) isolated from onions commonly possessed *SIX3*, *5*, *7*, *9*, *10*, *12*, and *14*, whereas the strains isolated from Welsh onions harbored both *SIX2* and *SIX9* (AF17, AF22, AF90, and AF94), *SIX2* (AF74), *SIX9* (AF113), or no *SIX* genes (AF67 and AF97). Notably, according to our results of the inoculation test for Welsh onion seedlings, all strains except AF97 were pathogenic to Welsh onions. This indicates that the AF97 strain is opportunistic toward Welsh onions. Among the strains used in this study, AF17, 22, 90, and 94, which have the *SIX2* and *SIX9* genes, caused relatively uniform severe disease in Welsh onion seedlings. Furthermore, the *SIX2* and *SIX9* genes are located on a single 3 Mb chromosome in the Welsh onion-infecting *Fusarium* strain [26]. It was speculated that the 3 Mb sized chromosome containing the *SIX2* and *SIX9* genes was evolutionarily acquired from other strains or that strains constructed the 3 Mb sized chromosome themselves, finally leading to pathogenicity toward Welsh onion. However, we could not clarify the obvious relationship between pathogenicity toward Welsh onion and the *SIX* genes in this study because the AF67 strains were pathogenic toward Welsh onion but did not harbor any *SIX* genes, and the gene expression of *SIX2* was dependent on the strain and host. Thus, further investigation of various *Fusarium* strains isolated from diseased Welsh onions would be helpful in understanding pathogenicity differentiation and the relationship between pathogenicity toward Welsh onion and the *SIX* genes. Moreover, intriguingly, pathogenicity differentiation toward Welsh onion was observed between AF67 (pathogenic) and AF97 (non-pathogenic), strains which lack *SIX* genes, suggesting that another pivotal factor other than *SIX* genes exists in pathogenicity toward Welsh onion. Therefore, a comprehensive comparison between AF67 and AF97 with several aspects, including biological characteristics, genome structure, and gene expression, would be desirable to deeply understand the pathogenicity toward Welsh onion.

Inoculation tests for onion bulbs and Welsh onion seedlings revealed that only the strains (AC145, AP117, Ru-13, and TA) isolated from onions were highly aggressive to onion bulbs, whereas all isolates, except for AF97, caused wilt symptoms in Welsh onion seedlings. It is plausible that onion-infecting *Fusarium* strains evolutionarily acquired the *SIX3*, *5*, *7*, *9*, *10*, *12*, and *14* genes for enhanced onion pathogenicity. Previous studies [5,35] have reported that *Fusarium* species strains containing *SIX3* and *SIX5* were relatively more aggressive to onions than those not containing these *SIX* genes, which is consistent with our results. All *SIX* genes (*SIX3*, *5*, *7*, *9*, *10*, *12*, and *14*) in the onion-infecting Japanese TA strain are located on a single pathogenicity chromosome [22]. Therefore, it was speculated that the *SIX* genes (*SIX3*, *5*, *7*, *9*, *10*, *12*, and *14*) possessed by strains isolated from onions are evolutionarily acquired via horizontal chromosome transfer to enhance the pathogenicity toward onions. Notably, the strains isolated from onions in this study were collected from Japan, Australia, The Netherlands, and Russia. Moreover, the strains (AC145, AP117, Ru-13, and TA) exhibited a common *SIX* gene profile and caused severe disease in onion bulbs, implying the global distribution of the clonal pathogenic *Fusarium* strains. Indeed, the Japanese TA strain used in this study exhibited high genome similarity to the pathogenic British *Foc*_FUS2 strain [22], further supporting our findings.

Here, inoculation tests for onion bulbs and Welsh onion seedlings revealed that the strains harboring the *SIX9* gene were highly aggressive to at least either onion or Welsh onion, indicating the importance of *SIX9* in pathogenicity. Gene expression of *SIX9* is upregulated during infection and associated with the degree of pathogenicity toward onions [24,35,36]. Here, the *SIX9* gene was expressed during infection of both onions and Welsh onions, suggesting its role in the pathogenicity toward onions and Welsh onions. Moreover, the *SIX9* gene might be involved with a common invasion function in various plant species rather than host-specific pathogenicity. Indeed, in the *F*. *oxysporum* spp., *F*. *oxysporum* f. sp *cubense*, *lycopersici*, *narcissi*, *niveum*, *palmarum*, *pisi*, *radicis-cucumerinum*, *raphani*, *sesame*, *spinaciae*, *Arabidopsis*-infecting *F*. *oxysporum*, and non-pathogenic *F*. *oxysporum* possess the *SIX9* gene [18,21,35,36,37,38,39,40,41]. Recently, gene knockout and gene interference techniques have been used to reveal the functions of genes. Therefore, further research on the *SIX9* gene using gene knockout and gene interference techniques is necessary to clarify the specific roles of the *SIX9* gene in determining pathogenicity. If the *SIX9* gene is related to pathogenicity toward *Allium* spp., the *SIX9* gene could be a useful molecular marker for diagnosis in infected soil to detect the highly pathogenic *Fusarium* strains toward *Allium* spp.

In this study, we clarified that the *Fusarium* strains previously described as *Foc* were placed in the clade of *F*. *nirenbergiae* and *Fusarium* sp. in the newly established epitype of *F*. *oxysporum* and provided insight into pathogenicity differentiation in *Fusarium* spp. toward *Allium* spp. Further comprehensive investigation would disclose the pivotal factor required for pathogenicity in *Fusarium* spp. toward *Allium* spp. and this information would be a useful tool for effectively conducting disease management and providing an understanding of the molecular mechanism of pathogen–host interaction between *Fusarium* spp. and *Allium* spp.

## 5. Conclusions

In summary, the strains previously identified as *Foc* were classified into *F*. *nirenbergiae* and *Fusarium* sp., but not *F*. *oxysporum* sensu stricto, via phylogenetic analysis of the combined sequences of *cmdA*, *rpb2*, and *tef1*. Furthermore, to verify the host specificity of the strains isolated from onions and Welsh onions, inoculation tests were performed on onions and Welsh onions as potential hosts. The results revealed the diverse host specificity of the *Fusarium* strains isolated from onions and Welsh onions. The inoculation tests also provided insights into the evolutionary pathogenicity differentiation of the strains causing Fusarium basal rot and wilt disease. We hypothesized that the non-pathogenic *Fusarium* strain might have evolved to become pathogenic in Welsh onions via the vertical or horizontal acquisition of pathogenicity-related factors. Subsequently, the strain likely evolved pathogenicity toward onions via the acquisition of pathogenicity chromosomes containing *SIX* genes. As only 12 strains were investigated in this study, more strains should be analyzed in future studies to verify our hypothesis. Nevertheless, this study would provide more certain proof or insight into pathogenicity differentiation in the causative agent of Fusarium basal rot and wilt disease in *Allium* species.

## Figures and Tables

**Table 1:** *Fusarium* species isolated from the diseased onion bulbs and Welsh onion seedlings in this study.

			*SIX* Gene								Accession Number ^a^		
Strain	Host	Origin	2	3	5	7	9	10	12	14	*cmdA*	*rpb2*	*tef1*
AC145	Onion	The Netherlands	−	+	+	+	+	+	+	+	LC795549	LC795537	LC795525
AP117	Onion	Australia	−	+	+	+	+	+	+	+	LC795540	LC795527	LC795524
Ru-13	Onion	Russia	−	+	+	+	+	+	+	+	LC795541	LC795526	LC795523
TA	Onion	Japan	−	+	+	+	+	+	+	+	LC795548	LC795536	AB938076
AF67	Welsh onion	Japan	−	−	−	−	−	−	−	−	LC795543	LC795532	AB938030
AF74	Welsh onion	Japan	+	−	−	−	−	−	−	−	LC795544	LC795530	AB938032
AF97	Welsh onion	Japan	−	−	−	−	−	−	−	−	LC795546	LC795533	AB938043
AF113	Welsh onion	Japan	−	−	−	−	+	−	−	−	LC795547	LC795528	AB938047
AF17	Welsh onion	Japan	+	−	−	−	+	−	−	−	LC795542	LC795529	AB938024
AF22	Welsh onion	Japan	+	−	−	−	+	−	−	−	LC795538	LC795531	AB938025
AF90	Welsh onion	Japan	+	−	−	−	+	−	−	−	LC795545	LC795534	AB938037
AF94	Welsh onion	Japan	+	−	−	−	+	−	−	−	LC795539	LC795535	AB938040

+ indicates the presence of amplicons; − indicates the absence of amplicons. ^a^ GenBank/DNA Data Bank of Japan (DDBJ) accession numbers for calmodulin (*cmdA*), RNA polymerase II second largest subunit (*rpb2*), and translation elongation factor 1 (*tef1*) determined in this study.

## Data Availability

Sequences of calmodulin (*cmdA*), RNA polymerase II second largest subunit (*rpb2*), and translation elongation factor 1-alpha (*tef1*) in each strain used in this study were deposited in DNA Data Bank of Japan (DDBJ).

## Supplementary Materials

The following supporting information can be downloaded at: https://www.mdpi.com/article/10.3390/pathogens13070591/s1, Figure S1: Gene expression of SIX2, SIX3, SIX5, and SIX9 during infection of onions and Welsh onions; Table S1: Primers used in this study. References [42,43,44,45,46,47] are cited in the supplementary materials.
