# Supplementary material for: Pathogenicity Differentiation of Fusarium spp. Causing Fusarium Basal Rot and Wilt Disease in Allium spp
Source: Pathogens. 2024 Jul 16;13(7):591. doi: 10.3390/pathogens13070591 (PMC11279435; doi:10.3390/pathogens13070591)
Supplement: Supplementary file 1 [file pathogens-13-00591-s001.zip › Figure S1.pdf]

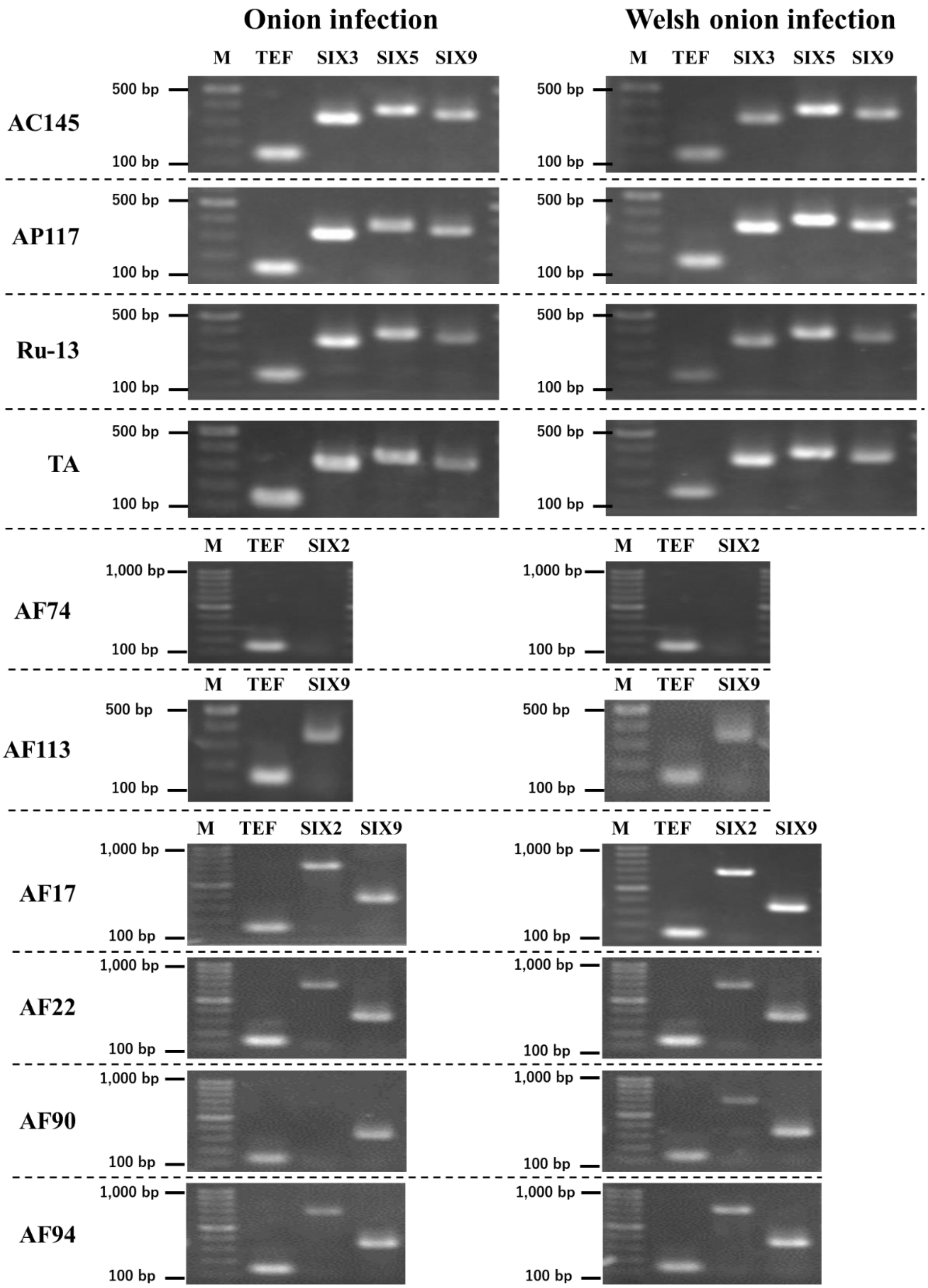

Figure S1. Gene expression of *SIX2*, *SIX3*, *SIX5*, and *SIX9* during infection of onions and Welsh onions. *TEF* gene is used as positive control.
