# Supplementary material for: Pathogenicity Differentiation of Fusarium spp. Causing Fusarium Basal Rot and Wilt Disease in Allium spp
Source: Pathogens. 2024 Jul 16;13(7):591. doi: 10.3390/pathogens13070591 (PMC11279435; doi:10.3390/pathogens13070591)
Supplement: Supplementary file 1 [file pathogens-13-00591-s001.zip › Table S1.pdf]

Table S1. Primers used in this study

Primer name	Sequence (5′–3′)	purpose	Reference
CAL-228F	GAGTTCAAGGAGGCCTTCTCCC	Sequencing for *cmdA*	Carbone & Kohn (1999)
CAL2Rd	TGRTCNGCCTCDCGGATCATCTC	Sequencing for *cmdA*	Groenewald et al (2013)
EF-1	ATGGGTAAGGARGACAAGAC	Sequencing for *tef1*	O'Donnell et al. (1998)
EF-2	GGARGTACCAGTSATCATGTT	Sequencing for *tef1*	O'Donnell et al. (1998)
5f2	GGGGWGAYCAGAAGAAGGC	Sequencing for *rpb2*	Reeb et al. (2004)
7cr	CCCATRGCTTGYTTRCCCAT	Sequencing for *rpb2*	Liu et al. (1999)
SIX2-F	TCCTCAACGGCGCATTAGTC	Detection of *SIX2*	This study
SIX2-R	GCAAGATGACTGTGAACCCAC	Detection of *SIX2*	This study
SIX3-C-F	CATGTCCATGACATGGGTTTGC	Detection of *SIX3*	Sasaki. (2015)
SIX3-C-R	GATCTCGTAGTTGGCGATGGC	Detection of *SIX3*	Sasaki. (2015)
SIX5-C-F	GCGCTTCGAGTACATCTCTG	Detection of *SIX5*	Sasaki. (2015)
SIX5-C-R	CTAGGATGCATCACAATAGA	Detection of *SIX5*	Sasaki. (2015)
SIX7-Q-F	CTTTTCCATTTCGCCCTGTTT	Detection of *SIX7*	Sakane et al. (2024)
SIX7-Q-R	TGGTGTTGAGCCGGTGCTA	Detection of *SIX7*	Sakane et al. (2024)
SIX9-F	TCAGCAGTTGCGGCAATGAC	Detection of *SIX9*	This study
SIX9-R	CACAGTGCATTGTCCCATCTC	Detection of *SIX9*	This study
SIX10-Q-F	TTTGCTCAAGGCTTCAACCA	Detection of *SIX10*	Sakane et al. (2024)
SIX10-Q-R	CGCGGTCCCGTAGATTAGTC	Detection of *SIX10*	Sakane et al. (2024)
SIX12-Q-F	GTTCTCGCCCAAAGCCATT	Detection of *SIX12*	Sakane et al. (2024)
SIX12-Q-R	TCACCTCCGGCTCGTAGTT	Detection of *SIX12*	Sakane et al. (2024)
SIX14-Q-F	CTTGGATGTCGTATGCCGGA	Detection of *SIX14*	Sakane et al. (2024)
SIX14-Q-R	GCCTTCCTTACCTTGGACCC	Detection of *SIX14*	Sakane et al. (2024)
FoTEF-Q2-F	CATCGGCCACGTCGACTCT	Detection of *TEF*	van der Does *et al*. (2008)
FoTEF-Q2-R	AGAACCCAGGCGTACTTGAA	Detection of *TEF*	van der Does *et al*. (2008)

References

Carbone, I., Kohn, L. M. A method for designing primer sets for speciation studies in filamentous ascomycetes. *Mycologia*, **1999**, *91*, 553-556.

Groenewald, J. Z., Nakashima, C., Nishikawa, J., Shin, H. D., Park, J. H., Jama, A. N., Crous, P. W. Species concepts in Cercospora: spotting the weeds among the roses. *Studies in Mycology*, **2013**, *75*, 115-170.

Liu, Y. J., Whelen, S., Hall, B. D. Phylogenetic relationships among ascomycetes: evidence from an RNA polymerse II subunit. *Molecular biology and evolution*, **1999**, *16*, 1799-1808.

O'Donnell, K., Kistler, H. C., Cigelnik, E., Ploetz, R. C. Multiple evolutionary origins of the fungus causing Panama disease of banana: concordant evidence from nuclear and mitochondrial gene genealogies. *Proceedings of the National Academy of Sciences*, **1998**, *95*, 2044-2049.

Reeb, V., Lutzoni, F., Roux, C. Contribution of RPB2 to multilocus phylogenetic studies of the euascomycetes (Pezizomycotina, Fungi) with special emphasis on the lichen-forming Acarosporaceae and evolution of polyspory. *Molecular phylogenetics and evolution*, **2004**, *32*, 1036-1060.

Sakane, K., Akiyama, M., Jogaiah, S., Ito, S. I., Sasaki, K.. Pathogenicity chromosome of *Fusarium oxysporum* f. sp. *cepae*. *Fungal Genetics and Biology*, **2024**, *170*, 103860.

Sasaki. Genetic lineage and pathogenicity genes in *Fusarium oxysporum* f. sp. *cepae*. **2015** , Doctoral course thesis.

van der Does, H. C., Duyvesteijn, R. G., Goltstein, P. M., van Schie, C. C., Manders, E. M., Cornelissen, B. J., Rep, M. Expression of effector gene *SIX1* of *Fusarium oxysporum* requires living plant cells. *Fungal Genetics and Biology,* **2008**, *45*, 1257-1264.
